# Quantum Collapse and Computation in an Everett Multiverse

**DOI:** 10.3390/e26121068

**Published:** 2024-12-09

**Authors:** Fabrizio Tamburini, Ignazio Licata

**Affiliations:** 1 Rotonium, Le Village by CA, Pz. G. Zanellato, 23, 35131 Padua, Italy; 2 Institute for Scientific Methodology (ISEM), 90156 Palermo, Italy; ignazio.licata3@gmail.com; 3 School of Advanced International Studies on Theoretical and Nonlinear Methodologies of Physics, 70124 Bari, Italy; 4 International Institute for Applicable Mathematics and Information Sciences (IIAMIS), B.M. Birla Science Centre, Adarsh Nagar, Hyderabad 500 463, India

**Keywords:** quantum measurement, semantic closure, von Neumann universal constructor

## Abstract

The mathematical representation of the universe consists of sequences of symbols, rules and operators containing Gödel’s undecidable propositions: information and its manipulation, also with Turing Machines. Classical information theory and mathematics, ideally independent from the medium used, can be interpreted realistically and objectively from their correspondence with quantum information, which is physical. Each representation of the universe and its evolution are, in any case, physical subsets of the universe, structured sets of observers and their complements in the universe made with spacetime events generated by local quantum measurements. Their description becomes a semantically closed structure without a global object-environment loss of decoherence as a von Neumann’s universal constructor with a semantical abstract whose structure cannot be decided deterministically a priori from an internal observer. In a semantically closed structure, the realization of a specific event that writes the semantical abstract of the constructor is a problem of finding “which way” for the evolution of the universe as a choice of the constructor’s state in a metastructure, like the many-world Everett scenario, from a specific result of any quantum measurement, corresponding to a Gödel undecidable proposition for an internal observer.

## 1. Introduction

The concept of a universe is mainly used to represent the whole physical world as the totality of space and time with all the physical phenomena, like the fundamental interactions, constants, etc., in terms of events $E_{i}\left(x^{j}\right)$ and interactions between events (as local coincidences of events). In certain theories, the relationships between events generate space and time at large *scales* as emergent quantities [1,2]. An event $E(x^{i})$ in the universe from a classical relativistic point of view is described as a suitably smooth (vector) map between the space-time 4-position $x^{i}=\left\{x^{0},x^{1},x^{2},x^{3}\right\}$ and a suitable parameter space in which are locally defined the properties of the observables associated with the fundamental fields, their conserved quantities, currents and other invariants. To each event $E(x^{i})$, one associates the local present defined by its past, causally generating the future and the simultaneity of events, as summarized in the light-cone diagram. A special event is the space-time 4-position $x^{i};i=0,\ldots ,3$ itself. In special relativity, the local present is a point-event $E(x^{i})$ in the “*hypersurface of the present*”, made by all the events that are in the “*absolute elsewhere*” of $E(x^{i})$ that cannot affect or be affected by $E(x^{i})$. This hypersurface is orthogonal to the symmetry axis of the light cone. Any event and set of events together with their evolution is the result of the interactions of each of the subsets $E(x^{i})$ that build up the universe, according to Mach’s principle [3]. In general relativity, these interactions are obtained from the construction of local chains of observers and events when introducing the definitions of distances, time intervals and simultaneity [4]. In this framework, when mathematics is interpreted realistically and objectively [5] as the result of physical processes, our mathematical truths would result in metastructures of events in spacetime, built of coincidences and relationships of events, in disagreement with the belief that the “mathematical objects” should possess an a priori and unconditional reality that goes beyond the physical phenomena. Mathematical knowledge is not absolute, being that each mathematical formal structure is built from axioms and inference rules to be adapted for the description of any computational, physical or abstract scenario. In a certain sense, it would resemble a derivation from empirical knowledge, with given truths like axioms and inference rules presenting, in most cases, crucial limitations exposed by Gödel’s theorems with number theory, showing that the associated set of truths is not recursively enumerable even if it is expected that all the statements that can be proved from the axioms form a recursively enumerable set. Deutsch [6], from the work by Church [7], Kleene [8] and Turing [9,10], suggested a deep connection between experimental physics with computer science and a universal quantum computer, and that there would always exist a program for each physical process in Nature and that any program can correspond to a physical process.

Quantum Mechanics (QM) is open to the observer because of the unpredictability of the collapse. Here, we consider the wave function as a “reservoir” of possible outcomes and analyze the counterfactual classes and their contribution in the evolution of a physical system through Feynman’s paths. In the case of the wave function of the universe, we can therefore speak of an Everettian evolutionary scenario (according to the Hartle–Hawking formalism in an Everett scenario).

The universe and its evolution can be described by a pure quantum state Ψ(t), which is represented as a vector in the Hilbert space of the energy shell of the system. Equivalently, they are described in terms of a semantically closed structure that describes interactions between its subsets, sets of events, becoming a semantically closed structure without a global object-environment loss of decoherence. In the presence of decoherence, it is, in general, impossible to know exactly any outcome of a quantum measurement, as it depends on the choice of a set of unmonitored degrees of freedom, the environment or the complementarity of the observed system relative to the observer in the universe. It is, however, possible in principle to verify quantum-mechanical predictions exactly in a multiverse Everett scenario, “a single observer within the universe can access in infinitely many identical experiments; and the outcome of each experiment must be completely definite” [11].

In this case, a semantically closed structure describes a system that can enclose its meaning within itself. Following [12,13], in a semantically closed structure of sequences of symbols, the universe interacts with the description of itself at a given point of its symbolic sequence to replicate and evolve by copying the information encoded in its abstract description (its state and evolution at a given point of the sequence) to construct itself and its evolution with the set of rules there encoded. The sequence of symbols and rules that defines the information in the abstract description is itself encoded in that abstract description with its evolution.

In this structure, each of these subsets concur as constructors or parts of the constructor for the evolution and existence of subsets up to the whole universe by their reciprocal interaction through coincidences of events as in von Neumann’s constructor [14].

These relationships are expressed with our basic mathematical and logical language, in terms of rules, concepts, axioms and theorems in order to build a representation of nature, whether the physical universe is finite, infinite or belongs to a Multiverse as a subset including Gödel’s undecidability theorems [15] that arise when one adopts a complete mathematical description of the universe in terms of numbers and relationship between numbers, as initially proposed by Dirac [16], that are equivalent to physical systems in a universal quantum Turing Machine [6,17,18,19,20].

Many attempts have been made to relate the problem of a quantum measurement with Gödel’s undecidability (see Section 2.1), whether a proposition is true, false or undecidable, comparing the limits in the formal language with the nonclassical properties of the quantum world. From our assumptions, the measurement process is, in any case, a physical process in the universe and a key to its evolution described with a sequence of symbols. If the universe is a pure state, any measurement would affect the state of the universe and evolve it, leaving it as a pure state, mixing the states between observer and observed, introducing decoherence to the observed that can be the complementary of the observer in the universe.

The state remains a pure state if the observer is the empty set. Thus, the evolution of the universe depends on the interaction between its subsets and the problem of a measurement with a classical observer, viz., translating the evolution in terms of a formal classical language finds “which way” for its evolution in a Many-Worlds Everett scenario as a classical Gödel undecidability proposition formulated inside the substructure universe [21,22,23] and depends only on the mathematical language and modeling used to describe the problem that is being faced.

In this work, we will discuss the challenges posed by the correspondence between classical and quantum computation models of physical systems involving a multiverse approach to quantum mechanics understood through the relationship between the observing and observed systems in a universe, with some considerations on the limits of theories and the role of computability in describing the physical world. We focus on the Everett-type multiverse, also known as the Many-Worlds Interpretation (MWI) of quantum mechanics. This interpretation posits that all possible outcomes of quantum measurements are realized in a vast, branching multiverse, where each possible outcome occurs in its own distinct branch. Gödel’s theorems and a toy model for this discussion are reported in the Appendix A.

## 2. Computing in a Universe

Excluding non-recursively enumerable languages and problems like the Halting Problem, any formal language can, in principle, be represented by a Turing machine [24,25]. Gödel’s incompleteness theorem highlights undecidable propositions within formal arithmetic systems, which translates into computation through the Turing Machine Halting Problem and the Church–Turing thesis. When applying these concepts to physical systems, the nature of undecidability takes on new aspects, particularly in the axiomatization of physical theories that blend theoretical constructs with empirical observations [26]. Physics, despite its formalizations, is inherently semantic because every physical theory is grounded in operational procedures and experimental verification. Nonetheless, it is intriguing to explore whether physical theories contain undecidable propositions and what foundational questions a “theory of everything” might pose in this context. The Everett multiverse presents a compelling interpretation of quantum mechanics, suggesting that a theory of everything should remain consistent with empirical observations and established physics while embracing the deterministic evolution of the universal wave function [21,22,23]. Such a theory would aim to integrate Quantum Mechanics and General Relativity into a cohesive model that accommodates the branching nature of reality.

The Many-Worlds Interpretation of quantum mechanics provides a compelling framework by proposing that all possible outcomes of quantum events are realized in a vast, branching multiverse. In this interpretation, the universal wave function evolves deterministically, and the apparent randomness of quantum measurements arises from observers becoming entangled with the superposed states of the system. While concerns have been raised about the testability of such multiverse theories [27], the Everett interpretation remains internally consistent and offers the same predictive power as other interpretations without introducing additional unobservable mechanisms like wave function collapse. This work focuses on the theoretical exploration of undecidability within the Everett multiverse framework. In the Everett interpretation, certain propositions become undecidable due to the superposition of states and the branching nature of reality. For example, the exact outcome of a quantum measurement is not determined until an observer becomes entangled with the system, leading to different outcomes in different branches. From the observer’s perspective, this means that an observer within one branch cannot access or communicate with other branches, making propositions about the specific outcomes in other branches undecidable within their own branch. Consider a quantum system prepared in a superposition of states. Upon measurement, the system branches into multiple outcomes. For an observer in one branch, the proposition ‘The measurement resulted in outcome A in another branch’ is undecidable because they have no access to information about other branches. Within the formalism of the Everett interpretation, there is no mechanism for an observer to verify the outcomes in other branches, rendering such propositions undecidable (It is clear that the Everett many-worlds interpretation (EMWI) describes a multitude of branching realities that are thought to arise because of the possible results of a quantum measurement, while Feynman’s path integrals approach (FPI) describes the probability of the outcomes of a quantum measurement in terms of the sum over histories and focuses on calculating probabilities without needing the existence of distinct, branching worlds. EMWI relates the interpretation of the results of a quantum measurement by introducing branching. In FPI, instead, the sum over histories retains a single universe perspective and explains outcomes through interference of probabilities. While EMWI can be considered as an interpretation of all the possible scenarios described by quantum mechanics, the result of the sum over histories of FPI is a computational technique that can be applied within various interpretations that, when one considers a single possible outcome of the state of the universe after a quantum measurement, the result is, in the end, the same.

### 2.1. Undecidability and Uncomputability in Theoretical Physics

As is known, Gödel’s Theorems constitute a fundamental stage in the relationship between logic and mathematics. Shattering Hilbert’s formalist dream, the undecidability results contributed to the contemporary conception of mathematics as an open, non-zippable system [28]. Considering theoretical physics as a formal construction [29], it is interesting to investigate the possibility of finding undecidable propositions here too. In this sense, there are some general results that depend on the observer being immersed in the system he observes, as discussed in Refs. [30,31] and others more specifically addressed to the questions posed by quantum physics and cosmology, where the unpredictability of a event is rooted in the nature of things. Classical examples of foundational challenges in physics include the collapse of the wave function—which functions similarly to a ‘fifth postulate’ in quantum mechanics—and the cosmological configurations of the universe described by the Wheeler–DeWitt equation [32]. The Wheeler–DeWitt equation attempts to unify quantum mechanics and general relativity by formulating a wave function of the entire universe, encapsulating all possible cosmological configurations in a single quantum framework. However, a significant limitation of this approach is that it lacks a well-defined Hilbert space with an inner product structure. Without a Hilbert space inner product, it becomes impossible to define quantum expectation values for observables, which are essential for making physical predictions. This limitation suggests that while the Wheeler–DeWitt equation is a pioneering step toward a quantum theory of gravity, it may not fully satisfy the requirements of such a theory, as it cannot prescribe expectation values for all relevant quantum observables.

At the quantum level, the limitations of the Church–Turing thesis become evident due to the manifest incompleteness of quantum theory [6]. The Church–Turing thesis posits that any function computable by an effective procedure can be calculated by a Turing machine. However, quantum phenomena such as entanglement and superposition introduce non-local correlations and probabilistic outcomes that classical computational models cannot fully capture.

One way to describe this situation is that Shannon–Turing information, which is local in nature, cannot fully capture the detailed evolution of a quantum system, unlike in classical systems, due to the “hidden information” associated with quantum entanglement. This hidden information manifests through quantum potentials or Feynman path integrals, reflecting non-local correlations that are not easily computable using classical information theory. The problem becomes even more radical at the level of quantum gravity because, in General Relativity (GR), the causal structure of spacetime is dynamic and not fixed. When combined with quantum uncertainty, this leads to an indefinite causal structure [33,34,35,36]. This indefinite causal structure complicates the application of standard computational and physical theories, as they rely on well-defined causal relationships. Therefore, both the incompleteness of our current quantum theories and the limitations of classical computational frameworks suggest the need for new paradigms to fully understand and describe the fundamental nature of the universe.

Without a fixed background spacetime, we cannot define a global time parameter or a well-ordered sequence of events, and we cannot consistently determine the truth or falsity of statements about the sequence of events or the evolution of the system. As a result, certain propositions about the behavior of a quantum gravitational system become undecidable within the theory because there is no consistent way to compute or predict outcomes using existing algorithms or axioms. In other words, questions about the system’s evolution or state cannot be resolved as true or false due to the lack of a definitive causal order, leading to undecidable sentences in the theory of quantum gravity. In other words, standard computational methods and logical frameworks struggle to address these questions, highlighting fundamental limitations in our ability to fully describe the universe at the quantum gravitational level.

Undecidability arises in quantum mechanics independently of the multiverse concept. Within the Everett interpretation, propositions about outcomes in other branches or the experiences of alternate selves are undecidable due to the lack of interaction between branches. For instance, an observer cannot determine the outcome of a measurement in another branch, making such propositions undecidable within their own frame of reference. Additionally, standard quantum mechanics presents undecidable propositions due to quantum indeterminacy and the measurement problem. In quantum gravity, the combination of quantum uncertainty and the dynamic causal structure of general relativity leads to undecidable propositions regarding the sequence and causality of events [36].

Of particular interest for our purposes is to underline that the fundamental reason for the indeterminacy in quantum cosmology is of a mathematical nature and derives directly from the Gödel limits. It is the non-classification theorem of four-dimensional varieties; there is no algorithm that can classify all compact four-dimensional manifolds not limited, nor even capable of distinguishing between two of them [37]. This is, as is evident, an issue at the heart of the so-called “peaceful coexistence” between GR and QM and which makes it difficult to define a cosmological wave function. However, there are alternatives. For example, it is possible to choose for physical reasons a selection criterion that selects a geometry from the totality of the manifolds as a cosmological boundary condition. This, for example, is the path chosen by Hartle–Hawking and other physicists which assigns a special role to de Sitter’s geometry, recently confirmed by observations on the acceleration of the universe [11,38,39]. A more extreme solution could lie in the advent of cellular automata universe models on the Planck scale, and therefore, the extreme conceptual complexity of the varieties is not necessary if not coarse-grained, and is replaced by a discrete algorithmic complexity. This path was taken by ’t Hooft in an attempt to unite the interpretation of QM with particle physics [40,41]. ’t Hooft’s interpretation is often described as an attempt to reintroduce locality to the bottom of the QM, but this is not entirely accurate. In fact, the periodic orbits inside the cells which replace in a very precise sense the harmonic oscillators of QM cannot be observed directly (fast hidden variables), and the equivalence classes support the effects not local up to the Planck scale. It is, therefore, to all intents and purposes, an emerging version of QM. Even in this version, however, the measurement event seems afflicted by the unpredictable characteristics of collapse. Indeed, if we do not limit the idea of collapse to the traditional observer–observed binomial and connect it to the more general objective concept of interaction (a measurement is an interaction), we obtain an image of a universe that is “actualized” through interaction events unpredictable starting from the fundamental laws of physics, giving rise to emerging properties and metastructures and complexity.

One of the central issues in theoretical physics is the study of complex behaviors within systems. The notion of complexity is not singular; it spans across disciplines, justifying the multitude of possible approaches based on the peculiarities of the system under consideration. However, there is a deeper epistemological reason for this diverse landscape of the “archipelago of complexity”; it is the pivotal role of the observer in detecting situations of complexity, instances where the collective behaviors of a system lead to structural modifications and hierarchical orderings [34]. This consideration leads directly to the crux of the issue of emergence in physics.

In general, intrinsic emergence is when we see a discrepancy between the formal model of a system and the observed behaviors. In other words, the recognition of emergence expresses the necessity, or at least the utility, of creating a new model capable of encompassing the new observational ranges. This raises the problem of the relationship between different levels of description, leading to two possible situations.

The first is known as phenomenological emergence, which concerns the semantic intervention of the observer regarding the new behaviors of the system. It aims to create a model whose characteristics—selection of state variables and dynamic descriptions—are aimed at a more convenient description of the observed processes. In this case, it is always possible, at least in principle, to connect the two models through appropriate “bridge laws”, whose task is to link the two descriptive levels via a finite amount of syntactic information.

The second one is radical emergence, which involves a completely new and different description that cannot be linked to the original model. Here, a breakdown of the causal chain is usually observed and can be described with appropriate symmetries and irreducible forms of unpredictability. In this case, the connection between the theoretical corpus and the new model may require a different type of theory semantics, such as a new interpretation and a new arrangement of basic propositions and their relationships, as in statistical physics [42].

These two distinctions should be considered purely illustrative, as more varied and subtle intermediate cases can indeed arise. As an example of phenomenological emergence, consider the relationship between Newtonian dynamics and the concept of entropy (via Standish). Classical dynamics laws are time-reversible, whereas entropy defines an “arrow of time”. To bridge these two levels—the microscopic reversible dynamics and the macroscopic irreversible behavior—classical statistical mechanics employs Maxwell–Boltzmann statistics and probabilistic assumptions, which are centered on space-time symmetries (due to the isotropy and homogeneity of space-time, there are no privileged points, directions, or instants in a process of energy level de-correlation). This establishes a “conceptual bridge” between particle descriptions and entropy, thus connecting the microscopic and macroscopic analyses of the system. However, this connection does not cover all aspects of the problem and cannot be seen as a complete “reduction”. In fact, even within the framework of classical physics, entropy may locally decrease due to statistical fluctuations, and while the microscopic description provides fundamental insights, for practical purposes, we often describe a perfect gas using macroscopic parameters like pressure, volume, and temperature rather than tracking individual molecules [43].

Another example concerns EPR-Bell correlations and the role of non-locality in Quantum Mechanics. In the Copenhagen interpretation, non-local correlations are observed but are not part of the theory’s facts. In Bohm’s interpretation, the introduction of quantum potential allows incorporating non-locality within the theory. It is worth noting that historically, the EPR issue originated as an ‘ideal experiment’ between Einstein and Bohr on the ‘elements of physical reality’ of QM. Only later, with Bohm’s analysis and Bell’s inequality on the limits of local theories with hidden variables, was it possible to transform the issue into experimental matter. Neither Einstein nor Bohr actually expected to observe ‘spooky actions at a distance’. Importantly, the expression of non-locality in Bohm’s theory does not require additional formal hypotheses beyond the standard framework provided by the Schrödinger equations. However, while this new interpretative perspective provides a different understanding of the theory, it also raises issues regarding what has been termed the ‘peaceful coexistence’ between special relativity and QM.

In both briefly examined (cited) cases, we can see how phenomenological and radical aspects of emergence are deeply intertwined in the dynamics of the development of physical theories. Moreover, it underscores the fundamental role of the observer in modeling and interpretive choices. It is essential to note that the relationship between observer and observed is not a bipolar relationship and, to prevent epistemological impoverishment, it cannot be resolved in a single direction. Instead, it should be considered an adaptive process where the system’s internal logic meets our ways of acquiring information about it to construct theories and interpretations capable of providing a description of the system.

### 2.2. Computing the Universe as a Whole

The broader definition of the universe proposed is due to the medieval philosopher Iohannes Scotus Eriugena (810–877), who understood it as everything that is created and that is not created. For a modern mentality, the reference to what is not created, or cannot be created, is interesting in relation to the importance given to constraints. Today we could include in the definition the probability of an event imposed by Quantum Mechanics, and replace the theological accents with the big bang and the conditions that define space-time and physical laws. All this must be distinguished from the observable universe, whose boundaries are those we know from cosmology understood as the history of matter, and is different from the set of possibilities contemplated by theoretical physics. The push towards unification pushes the archipelago of physical theories towards a greater number of connections around some central islands (relativity, Quantum Mechanics) and some mathematical keys (gauge theories). These connections imply very strict requirements on the constraints of possibilities, to the point of suggesting the idea of a new approach to physics based on them [44]. The question we ask ourselves is whether any hypothetical theory of everything can be considered as a Gödel system and which aspects of the universe would remain undecidable or incomputable. It should be underlined that in the current state of knowledge, non-computability should not be understood in terms of algorithmic compression (Church–Turing thesis) because varieties constitute a central part of much of the knowledge of the physical world; furthermore, the very structure of quantum physics poses problems for the universality of a quantum Turing Machine [45,46]. An important ingredient of the universe is, in addition to chaos and randomness (Kolmogorov-type), the presence of organized complexity, which favors the development of structures with logical depth [47]. In the current state of affairs, it is very difficult to say whether this aspect of the universe derives from physical laws or rather from special boundary conditions, as seems more likely [48,49]. We have arrived at the crucial point regarding the question of the physical world as a Godelian system, understood in a broad sense. On the one hand, we can resolve the issue of the incomputability of the cosmological boundary conditions (WdW equation) by choosing a specific geometry, as in the Hartle–Hawking case; in this case, the collapse of the wave function remains an unpredictable event with characteristics of randomness, an undecidable event on the basis of fundamental laws. As is known, the enigmatic aspects of the collapse dissolve in Everett’s many-worlds interpretation, which has merged today with the cosmology of chaotic inflation [21,50,51]. This new powerful cosmological interpretation of QM seems capable of solving both of the two undecidability problems, that of the choice of boundary conditions for the universe and the collapse of the wave function, suggesting that the multiverse is a logically closed and consistent system fusing physical laws with boundary conditions [52].

The Church–Turing–Deutsch principle (CTD), formulated by Deutsch [6,53,54], states that a universal computing device can simulate any physical process; *“every finitely* realizible physical system can be perfectly simulated by a universal model computing machine operating by finite means”. Any Turing Machine can in principle be built to describe any physical phenomenon. Turing Machines, including quantum and classical computers, are, in any case, also physical systems and anything they can do is dictated by the laws of physics, including our language and the building of mathematical truths. Thus, the physical limits of computation are determined by the fundamental constants of Nature such as the speed of light *c*, the quantum of action *h* and the gravitational constant *G*, with well-defined quantitative bounds [55].

Paradoxically, following the laws of computation, ideally, the universe could be simulated by a quantum computer or by a suitable Turing Machine with the consequence that one can deduce the possibility that we could be the result of a quantum computer simulation and live inside it.

Differently from the mathematical languages in which can be defined true and false and undecidable propositions, these concepts take on radically different aspects in the axiomatization of physical theories, where the axiomatic approach has always been little more than an attempt at synthesis, mixing theoretical elements and empirical assumptions [26]. The point is that physics, however formalized, is never a syntactic system because every physical theory has an intrinsic semantics defined with operational procedures. Nonetheless, it is interesting to ask whether physical theories are undecidable and what foundational questions a “theory of everything” can pose.

One of the main points is the problem of the quantum measurement, which is at the heart of quantum information processing and is one of the criteria for quantum computation.

These properties also include metaproducts or emerging structures from sets of physical systems with emerging laws different from the basic laws of Quantum Mechanics. An example are Classical systems that deal with the concept of real numbers, which cannot be simulated by a Turing Machine, as a TM can only represent computable reals as the product of a finite calculation.

If the universe is finite, contained within a given finite region of space like in a sphere of radius *R*, it contains a finite amount of information and energy *E* and thus of entropy. This is given by the Bekenstein bound, an upper limit on the thermodynamic entropy:

(1) $$S\leq \frac{2\pi kRE}{\hbar c\ln2},$$

where *k* is Boltzmann’s constant, *ℏ* is the reduced Planck constant, and *c* the speed of light.

The entropy can also be described in terms of the Shannon entropy:

(2) $$H=\frac{S}{k\ln2}.$$

In other words, this quantity gives the maximal amount of information required to fully describe any given physical system down to the quantum level.

The information of a physical system, or the information necessary to perfectly describe that system, must be finite if the region of space and the energy are finite, as expressed in Equations (1) and (2).

In computer science, this implies that there is a maximal information-processing rate, Bremermann’s limit [56,57,58,59], for a physical system that has a finite size and energy, and that a Turing Machine with finite physical dimensions and unbounded memory is not physically possible. Unless we assume that the mathematical truths are emerging metastructures, viz., structures that do not directly depend on the initial physical laws, the actual representation of integer and real numbers would not be possible in a finite universe, including the representation of infinities, unless we assume the existence of local continuous variables that reflect the classical concepts of space and time or the quantum mechanical continuous variables. Being continuous, in the mathematical language, one needs to build an axiomatic definition that includes Dedekind’s cuts, with elements that have to be Dedekind-complete [60] or with Tarski’s axiomatization [61], that do not show a direct connection with what we call the basic physical principles.

Following the Constructor theory, information is expressed in terms of which transformations of physical systems are possible and which are impossible using the language of ergodic theory [62]. An input substrate is processed by the Constructor giving an output substrate. Each event or measurement process can be expressed in terms of constructors and substrates up to a universal constructor for which input and output substrates represent the evolution of the universe, namely a universal constructor. In this way, the universe can be represented in the Constructor theory framework. Being a product of the mathematical language, a Constructor to represent the whole universe must have the same set of information to build and build the evolution of the universe. Information is something whose nature and properties are determined by the laws of physics alone. Information is also of the essence in the preparation of a state and measurement in physics; input and output of state preparation and measurements is represented by a set of information quantities, with information being a physical process.

Modern physics has recently developed some high-level phenomenology models, setting a sort of end-game theory model, which needs no a priori notions, to obtain a way of describing the system universe as a whole, like in the Wheeler–De Witt wave function of the universe, in the view of describing the universe in terms of a self-consistent semantically closed object.

Axiom-based models generate object-based logic and metastructures with their composition meta-rules, and are based upon symbols acting as fictitious objects obeying some meta-rules, requiring an infinite hierarchical regress to higher-level modeling, capable of building from any emergent phenomenon. This is an aspect derived from Gödel theorems for a formal logical system that can be, under certain hypotheses, classically translated into a Turing Machine by the classical Church–Turing thesis; each computable function can be computed by a universal Turing Machine. While generalizing this universality to quantum computation, we recall that there should be a universal quantum Turing Machine performing any desired unitary transformation on an arbitrary number of qubits, including a program as another part of the input state, or the program effecting a unitary transformation is independent of the state of qubits to be computed. It is shown, however, due to entanglement, that neither of these two situations exists in Deutsch’s quantum Turing Machine [6]. In this case, an input state is unitarily evolved to an output one. Such an algorithm, written in classical language, consists mainly of two parts:

1. How to embed the problem in the input state and the result in the output state.
2. How to realize the desired unitary transformation in terms of various quantum gates and wires, i.e., how to construct a quantum computational network. As shown in [63], the Church –Turing thesis cannot be, as it is, generalized to quantum computation, i.e., an arbitrary unitary transformation can be realized by a network composed of repeated application of local operations of gates and the algorithm for composing the network is classical. We have two types of universality in its quantum generalization:
  *Type-I* universality refers to the ability of performing any desired unitary transformation on an arbitrary number of qubits, by including a program as another part of the input state, similar to the classical one.
  *Type-II* universality means that the same program can be used for different input data.

Linearity and unitarity of quantum evolution conflict with these two types of universality in Deutsch’s QTM, and the two types of universality in quantum computation, as possible generalizations of the notion of universality in classical computation, as stated in the Church–Turing thesis, do not exist, because with dynamics fixed, linearity and unitarity of quantum evolution makes it impossible to synchronize different quantum paths of any possibility. This difficulty originates in entanglement. For a specific quantum computation, however, there is no such difficulty by definition; the Church–Turing thesis is interpreted as a physical principle, related to the problem of quantum measurement.

To model without a priori notions and infinite nesting into metastructures, start-up axioms must be given via a universality property by self-organized criticality. This describes the property of many systems to self-organize in such a way that the system itself moves towards states characterized by a fractal-like description, with no fundamental local scale. It was shown in [64], generalizing Gödel and Chaitin results in mathematics, that self-referential and self-contained systems, such as the universe, must involve intrinsic non-local randomness, namely self-referential noise as a realization-independent characterization of self-referencing. Recent developments in nonlinear quantum field theory, also according to prescriptions of Nelson’s Quantum Mechanics and stochastic quantization, have also recently shown that a simple SO(10) model could generate stochastic behavior during its evolution [65], giving universality to the chaotic inflationary scenario described via an interacting many-field model. This “noisy” property is a general feature of the quantum scenario; Wiener processes and Fractional Brownian Motions characterize each model evolution with a specific fractal behavior depending on its global particle statistics in a generalized Haldane scenario [34,66]. Chaitin stated that if the system is sufficiently complex, the self-referential capability of arithmetic results in randomness and unpredictability from a thermodynamical point of view. Local randomness also arises from quantum measurement processes, which is shown to be an undecidable proposition inside the structure universe, and should require a metastructure as an Everett scenario in which it must be defined as a universe-choice process following the model of Nelson’s Quantum Mechanics [67,68]. Using a classical formulation produced from nonlinear dynamics, in a finite time computation model (i.e., locally), quantum and classical nonlinear system are indistinguishable, and the choice corresponds to an arbitrary stop of a classical Turing Machine.

Heisenberg’s uncertainty principle in this scenario could act as a geometric self-similarity operator, and the Mach principle is intended as a measurement operator, essentially given by the nonlinearity property of the fields acting as a semiclassical object interacting with a quantum one (as happens during reheating during inflation, described semiclassically because it is interacting with other quantum scalar fields). So, the self-reproducing universe produces its existence by its own self-interaction. Showing that the problem of quantum measurement is closely related to Gödel’s thesis, this could imply as a *necessary* condition the existence of a metastructure, or of a self-referential structure given by a self-reproducing and organized hyerarchical interacting baby-universe scenario, geometrically described as a scaling characteristic of its fractal property by the Heisenberg uncertainty principle: Noether’s theorem. The (self)generation processes of the universe are such as to guarantee a quantum tessellation (with the Generalized Uncertainty Principle) and then an emergence of what we call isotropic and homogeneous spacetime. There is no clear difference between the universe and the multiverse, especially in QM (The concepts of “inside” and “outside” depend on the degree of logical openness of the model, which cannot be infinite, i.e., what is valid for mathematical metastructures does not necessarily apply to physical events, which also gave rise to formalisms. Among other things, the development of quantum gravity will lead us to better define these aspects suspended between continuous and discrete).

An example can be found in the inflationary scenario; Linde and Vilenkin [50,69,70,71] discussed the possibility, under certain hypotheses, of the existence of a self-reproducing inflationary universe described as a self-reproducing fractal structure. In this context, this scenario could be successfully seen as belonging to the class of what we call a “meta Everett Universe”, without needing a specific initial choice of parameters, interpreted as an emerging self-semantically closed structure which reproduces itself during its evolution.

Some properties of the language able to discuss this sort of semantically closed self-objects are classically well described by second-order cybernetics concepts; as an example, von Foester [72,73] postulated the existence of solutions for an indefinite classical recursive equation derived from a Piaget recursive structure of implications, which describes an observer’s account of a causal interaction between observer and observed without any starting point and with event ordering property, i.e., a chain of implications into the self-referential structure that defines ordering processes inside the structure, as stated by von Neumann. The solutions to this symbolic equation represent a stability structure in terms of discrete eigenvalues $O_{i}$ into the chain of infinite implications and act in the formal logic structure as a group of axioms for a metamodel, i.e., a model of modeling the reality, in which all fundamental properties can emerge from a self-organizing process of the structure universe itself, by means of a process called eigenbehavior. A self-organizing system like this is autonomous *iff* (if and only if) all structural processes that define and sustain all its dynamics are internally produced and reproduced, with an organizational closure, as seen above. Changing its classification ability, it is shown that the system must change its own structure. Following this prescription, it is possible to define the problem of quantum measurement as a process, i.e., an object inside and belonging to the universe itself, the result of an ensemble of physical processes that concur to create a global one which changes its own structure, defining a cosmic time, keeping the energy constant, according to thermodynamics and the decoherence problem of a pure state (in fact, if we make a distinction between observer and observed, the transformation of such a pure state as the universe into a mixed one by a measurement process is described by the change of Hamiltonian mean value, in violation of the energy conservation principle).

Thus, each physical process can act as a measurement process to its complementary into the universe, giving rise to a surging up of “infinite” state superposition, concurring to describe the evolution of the global state universe, as the result of a collapse of all those possible states of existence. The problem with infinities is so linked with Cantor’s theorem, which denies bijections between N and R, or the classical Turing Machine stop for non-computable functions.

In this way, roughly speaking, the collapse and the quantum measurement should be removed from the aura of “mystery” once and for all; in the end, we simply cannot count all the interactions in the universe (which we should have known since Feynman paths and Bohm’s potential [74]). With Everett (and in this part, our meaning is strengthened and clarified), Rovelli and Von Foerster claim that we build our “eternal brilliant garland”, also removing the naïve residue of decoherence. Without collapse, there would be no particles! What remains, up to the conclusions, brings to fruition the link between physics and computation. In practice, we have removed Everett and decoherence from the banal readings of the “fundamentals”, bringing them back to the center of concrete physics. A rough synthesis could look like this. Every nucleation from the quantum vacuum, i.e., production of the universe, is so rich in complexity (viz., metastructures) that for every observer-language there will always be physically undecidable propositions.

In principle, this can be described by a Heraclitean Process System (HPS) with self-organizing critical characteristics: randomness, nonlinearity, nonlocality and iterative structure, to give rise to a fractal-like structure. The linear iterative map is given by the results of a local quantum mechanical measurement, generating self-referential noise. It is a manifestation of HPS characteristics via objectification processes, such as nonlocality configurations induced by macroscopic objects, or nonlinearity itself, such as Mach principle states. Nonlinearity behaves as a macroscopic semiclassical apparatus, giving discrete jumps. Some approaches to this statement had been performed by defining a quantum measurement as a sequence of binary quantum jumps caused by a macroscopic apparatus [75,76], avoiding the creation of a perpetuum mobile of the third kind. The perpetuum mobile is defined considering statistical operators for composed systems before and after a measurement, $\rho_{b}^{comp}=P_{\psi_{b}}^{comp};\;$$\rho_{a}^{comp}=\sum \omega_{i}P_{\psi_{a_{i}}}^{comp},$ the mean value of the energy is expressed in terms of the Hamiltonian H: $E=Tr\{\rho^{comp}H\}$ and the energy and entropy change are $\Delta E=\sum_{i}\omega_{i}\left(\Psi_{ai},H\Psi_{ai}\right)-\left(\Psi_{b},H\Psi_{b}\right);\;\Delta \sigma =-\sum_{i}\omega_{i}\ln\omega_{i}>0$. If an observable *A* is measured, this means that ΔE≠0 violating energy conditions. But by thermodynamics laws $\Delta E=Q+W=tr\left\{\left(\Delta \rho^{comp}\right)H\right\}+Tr\left\{\rho^{comp}\left(\Delta H\right)\right\}$ that gives for a jump ΔE=Q; but Q>0,Δσ>0 that is a self-heating system.

In this way, an additional structure of the spacetime is added, in terms of a hypersurface with a constant value of what is interpreted as cosmic time. In the Heisenberg picture, the state vector changes at each quantum jump, which corresponds to a spacelike hypersurface. Each quantum jump gives a disjoint hypersurface, causewise ordered as in the von Foerster equation: $S_{2}>S_{1}$ or $S_{1}>S_{2}$. A cosmic macroscopic time for the instantaneous wave function collapse is defined, and time receives, in this scenario, a precise meaning as any other quantum result. When applying the measure to the state universe, we must define an operator capable of describing the collapse of a state inside the Everett scenario. The description of whether a quantum measurement operation has occurred or not is given by Rovelli operator *M*, [77], which has the crude meaning of “*It has happened or not*” and logical eigenvalues “1” or “0”.

To give an example, consider a physical system whose observable can be usually expressed in terms of state projectors as:

(3) $$A=\sum a_{i}P_{i},$$

where *P* is the general projection operator on a given basis. In our case, we consider the universe in a split between an observer, a part of the universe and the complementary set in the universe of the observer from the point of view of the internal observer. Let us consider the system vector under measurement φ and ξ, the vector-state apparatus, then ψ represents the composed system between them after an interaction. Before the measurement process, we have $\psi_{bi}=\varphi ⊗\xi$, then the state becomes $\psi_{ai}=\varphi_{i}⊗\xi_{i}$ with probability $\omega_{i}=\left(\varphi ,P_{i}\varphi \right)$ and $\varphi_{i}=\frac{P_{i}\varphi }{∥P_{i}\varphi ∥}$.

From the definition in Equation (3), their statistical operators are defined in the following way:

(4) $$\begin{array}{c} \rho_{b}^{comp}=P_{\psi_{b}}^{comp}\\ \rho_{a}^{comp}=\sum \omega_{i}P_{\psi_{a_{i}}}^{comp} \end{array}$$

which correspond to the vector ψ of the composed system, in agreement with the energy conservation principle and with laws of thermodynamics. By the concept of decoherence, the pure state universe is seen as the composed system of Equation (4) and is equivalent to the one given after the measurement in terms of state projectors:

(5) ρacomp~=Pψacompψa=∑(Piφ)⊗ξi⇒ρacomp~=ρacomp

with the decoherence condition

(6) $$\left(\psi_{aj},O\psi_{ai}\right)=\left(\varphi_{i}⊗\xi_{i},O\varphi_{i}⊗\xi_{i}\right)=\delta_{ji}.$$

To avoid the decoherence condition of Equation (6) and obtain the pure state universe, following Equation (5), one has to make indistinguishable the pure state from the mixed one, embedding all into a structure that must evolve after each process has occurred, changing its structure in terms of interactions between its subsets.

We can state that each event concurs to the evolution of all universes and all universes generate each event, as the Mach principle states. This defines the universe in terms of a von Neumann’s universal constructor [14]. To give a simple example, the measure of *A* is described with two discrete eigenvalues only, e.g., $a_{1}$ and $a_{2}$ with eigenstates $|a_{1}\rangle$ and $|a_{2}\rangle$ and interaction system-device Hamiltonian $H_{I}$.

Following the prescriptions of Quantum Mechanics, we prepare the state of the device |init〉 such that in a finite time, the interaction will evolve into $|a_{1}\rangle ⊗|init\rangle$ into $|a_{1}\rangle ⊗|\xi a_{1}\rangle$ and $|a_{2}\rangle ⊗|init\rangle$ into $|a_{2}\rangle ⊗|\xi a_{2}\rangle$, i.e., $\psi_{a}=\omega_{1}\varphi_{1}⊗\xi_{1}+\omega_{2}\varphi_{2}⊗\xi_{2}$.

Then, $\psi \left(0\right)=(\omega_{1}|a_{1}\rangle +\omega_{2}|a_{2}\rangle )⊗|init\rangle ⟶\psi \left(T\right)=\omega_{1}\varphi_{1}⊗\xi_{1}+\omega_{2}\varphi_{2}⊗\xi_{2}$ is a pure state that is replaced with one of the two substates after the wave function collapse $\psi_{1}=\varphi_{1}⊗\xi_{1}$ or $\psi_{2}=\varphi_{2}⊗\xi_{2}$. Computing the probabilities for the collapse, we have a correspondent hypersurface for any given state ψ(t) of the combined state observer–observed (φ⊗ξ) system by means of Rovelli correspondent operator $M\equiv |\psi_{1}\rangle \langle \psi_{1}\left|+\right|\psi_{2}\rangle \langle \psi_{2}|$ acting in this way:

State “happened” with eigenvalue 1

$M\left(\varphi_{1}⊗\xi_{1}\right)=\varphi_{1}⊗\xi_{1}$ and $M\left(\varphi_{2}⊗\xi_{2}\right)=\varphi_{2}⊗\xi_{2}$

State “not happened” with eigenvalue 0

$M(\varphi_{1}⊗\xi_{2})$ and $M(\varphi_{2}⊗\xi_{1})$

In this way, time and measure and event take sense into the Everett’s Many-Worlds model, in which every instant of time corresponds to a special case of a universe, and a measure with its result is described as a causal process with an ordering operator capable of defining a cosmic time. Only in this meta-universe can the problem of decoherence be translated as a problem of universe choice; phase coherence is lost by physical decoherence into the environment, making pure and mixed state indistinguishable and changing the structure of the global state in terms of mutual relationships between its substates, which means time evolution. The complementary of an observer can in fact be seen as the environment surrounding a quantum system. In this way, the observer can monitor the observables, or part of them, of the system. The effect of the observer is to induce decoherence continuously in the eigenstates of these observables and can assume the behavior similar to that of classical or pseudo-classical states [78,79,80], as in the convergence of a quantum Turing Machine into the final state of computation.

For this reason, the Everett scenario seems to become a sort of necessary environment for the evolution of the pure state that represents the object universe when described in terms of classical (or pseudo-classical) formal propositions with the possibility of building a Gödel symbolic construction (or a Turing Machine) to describe the evolution of the universe and the interaction between subsets up to the interaction with “classical” (or pseudo-classical) observers, which leads to the problem of a quantum measurement to be related to an undecidable proposition inside the linguistic representation of the universe itself. The implication is to have as mandatory the existence of a metastructure as a “linguistic” meta-universe. Being that this representation of the meta-universe is a class of universes in the evolution of our universe and at the same time built with physical events in the universe, the class should be a subset of the universe by definition or coincide with the universe, recalling Russell’s paradox. The mandatory requirement of the existence of a universe of universes shows that our classical language is inadequate for describing the state universe as a whole self-object unless we employ a self-bootstrapped structure, in which the laws of physics self-sustain one another through their mutual consistency [81].

## 3. Conclusions

In our formal language, the universe can be described in different ways. Each single bit of information is the result of a physical process inside the universe. An example is an end game modeling a self-referential system with a semantically closed structure containing intrinsic randomness, as any of its representations is a subset where there is not a global object-environment loss of decoherence but interactions with its subsets, indicating that its evolution, including the mathematical truths, undecidable propositions and quantum measurement problem, are metastructures inside the universe built with interactions between subsets. Our formal language is based on mathematical truths and any formal mathematical modeling of the universe as a whole, as initially proposed by Dirac, inherits Gödel’s undecidability. If one adopts this approach, the evolution and the problem of measurement is deeply related to undecidable propositions and Everett’s Many-Worlds interpretation of Quantum Mechanics becomes a meta-structure to contain all the possible states of the past and future evolution, showing the limits of our formal language in the description of the universe as a whole, showing a clear difference between models of physical systems and a complete formal language. Following Ref. [51], the language of Many-Worlds Quantum Mechanics is different from that of Quantum Mechanics, where any event depends on the probability amplitude of the wave function of any possible event. In our case, if we have to adopt the Many-Worlds scenario, in this case, “the wave function is a relative density of universes in the multiverse amplitude”. This means that in Many-Worlds, the Born frequencies, related to the square of the absolute value of the wave function that gives the best estimate of the probability density, is caused by the intrinsic deterministic nature of the wave equation. This implies, as described by our deterministic classical language, that the evolution of quantum systems in the multiverse, the universe of universes, is described by a deterministic wave equation. Born frequencies are thus approached asymptotically, rather than being defined a priori, as occurs in the computation of the Quantum Turing Machine discussed before, which can be isomorphically related to a classical Turing Machine. From this, the nonlocality of Quantum Mechanics also has a different interpretation from that in Many-Worlds [82], suggesting that at Planck’s scale, there will be not a different behavior; any observer should be “quantum” or a classical singularity. Instead, from the properties discussed at those scales [83] and near the horizon of a black hole [84], this would lead unavoidably to a holographic scenario described by cellular automata [41], which founds an agreement with the Many-Worlds scenario when handled with our language and mathematical truths. Being that the description of the universe is built with mathematical truths, and identifying the set of possible universes of Everett’s scenario as a class containing each possible set of event subsets, this defines the universe as in Russell’s paradox. The only way is to postulate the independent existence of the so-called laws of physics and consider any pure mathematical modeling for the description of the universe incomplete. The limits of our language suggest that we cannot use it to simulate a universe, as it requires truths from axioms acting in terms of “outside” and followed by inference laws with theorems and undecidable propositions. The only way one could simulate the Universe is to use a semantically closed structure based on a quantum language that is not accessible for an observer (like us), as it acts with its complementary in the universe needing a free will axiom in Quantum Mechanics [85,86].

## Data Availability

Data is contained within the article.

## Appendix A

### Appendix A.1. A Short Introduction to Gödel’s Theorems

Let us introduce Gödel’s Theorem with a few words. There are many excellent expositions of Gödel’s undecidability theorems, and here we will limit ourselves to offering an intuitive illustration useful for introducing his projections on theoretical physics. At the beginning of the 20th century, Hilbert’s program on the axiomatization of mathematical theories triggered intense research to describe at least a particular class of formal systems, those that were sufficiently powerful, through a logical syntax. These are those systems that are something more than a simple logical “toy”, and which have a structural complexity at least equivalent to that of natural arithmetic. Practically all interesting formal theories, including physical–mathematical ones, fall into this category. Sufficiently powerful systems have a notable self-referential capacity, that is, they are able to produce propositions that concern their internal structure, on which their “fecundity” depends. This is the keystone of Gödel’s 1913 theorems [15], which set very precise limits to the Hilbert program, as it is developed, e.g., in the powerful “Principia Mathematica” by Russell and North Whitehead (1910–1913) [87], showing that formal systems “pay” for this great expressive capacity with logical complications of a radical nature:

**Gödel’s first theorem:** Every sufficiently powerful, coherent and axiomatizable system is syntactically incomplete.

This result expresses that it is always possible to produce, starting from a system of axioms A, an undecidable proposition P, i.e., of which it is impossible to establish, with the tools of the system, either the truth or the falsity. In the theoretical context offered by the Turing Machine, this is equivalent to the famous halting problem; there is no general program (algorithm) which, applied to a particular pair (program–argument), is able to establish a priori whether the relative computation pf the couple in question will end or not.

**Gödel’s second theorem:** Any sufficiently powerful, coherent and axiomatizable system is incapable of proving a proposition that canonically expresses the coherence of a system.

In a certain sense, the limitation of this theorem is even more drastic than the first. In fact, the theorem states that using the syntactic–formal tools of <L, A, R>, it is impossible to demonstrate the logical solidity of the system itself, and in particular, to predict the production of a contradictory development. The hope placed by Hilbert in the axiomatic method as an instrument for the security of a logical foundation of mathematical knowledge was thus undermined at its roots. Outside of formal languages, it is possible to understand that axiomatization procedures require a typically tree-algorithmic compression for each mathematical theory, which is certainly possible within the semantic world of each specific model. However, when the emphasis is placed on purely syntactic aspects, the specter of undecidability hovers over the production of new propositions because, contrary to what many people think, mathematicians do not only manipulate symbols but also meanings, building connections between different models with perfectly legitimate procedures; first-order propositional calculus is coherent, complete and decidable and generally not very risky. This means that the calculus of predicates, which is obtained from the propositional calculus with the introduction of quantifiers, is undecidable, but in general, the semantic constraints on the discourse they protect from logical flaws. In other words it is legitimate to think that if undecidability comes into play even with “elementary” systems, such as the axiomatizations of arithmetic, the situation can only become critical when developments that imply different areas are considered superimposed. A now-classic example is Fermat’s theorem, cited by Gödel in a 1928 conference as a possible undecidable proposition [88]. The theorem was then proved by A. Wiles in the 1990s using very different branches of mathematics, completely unknown in Gödel’s time [89]. A recent example is the proposed solution of the Riemann conjecture through the equation with infinite Majorana components (Majorana tower). In this case, the construction procedures of the demonstration also went far beyond the possible care in the construction of an axiomatic system; the two structures in fact belong to very different semantic fields [90].

### Appendix A.2. Language Toy Model for a Toy Universe

We now show with a simple toy model that the description of the universe in terms of Turing Machines and constructors with our mathematical tools and truths unavoidably leads to relating the events in the universe and its evolution with the undecidable prepositions in our language, following point-by-point the logical structure set by Gödel to define the undecidable propositions in a logical structure.

To proceed in this paradox, one of the further steps to perform is the generalization of the Turing Machine to describe continuous spectra, translating the construction of a hypersurface quantum jump in usual terms of either probabilistic or quantum computation. In this case, it is possible to build an integer-number codification of an obtained ordered sequence of numbers and numerals, which correspond to physical quantities and laws, seen as numerical relations defined in the structure of the universe itself. An example is the adimensional Dirac [16] construction of physics, where numbers express physical quantities and relationships between numbers represent laws:

(1) event, causally measured physical quantity as a number;
(2) laws, relationships between quantities, class → numeral;
(3) measure, coincidence or relationship between events, class → numeral.

What is observed, or physically defined, is the result of an indefinite succession of cognitive (cause–effect) interactions that describe all the possible subsplittings of the pure state universe in a superposition of mutually interacting subsets. To obtain a formal-logic description of the universe, the self-evolution of an object with ordered time as internal state must be described, and must use proper elements of the system to describe relationships between other elements belonging to the system inside the system itself, as a self-referential structure. According to Gödel’s undecidability theorem [15], it is impossible to show formal coherence of the structure inside the structure itself. The a priori choice of the result of a quantum measurement, expressed through the classical logical language, as discussed before, becomes an undecidable proposition inside the universe itself, and makes sense only in an Everett scenario. The problem of quantum measurement, as defined, becomes an undecidable proposition inside the structure of the universe itself; for this purpose, some tools, variables and classes are defined, as in [91]. This ensures that assertions are also valid for transfinite systems: the primitive concept of “*follows*” by operator *f*; −, ^~^ are “Not”, ∨ Or, ≡ equal by definition (x)≡∀ for each; ⊃⊂ iff; (x),(Ex),(εx) with a border for the variable x, are used in definitions and propositions to express that the concepts defined there are recursive.

Following any logical system as a physical model corresponds to a meta-(…)-metastructure of integer numbers *Z* going from a logical formulation of higher order, justifying certain undecidable propositions, and shortening in a pretty large way an ideally infinite number of other proofs, but they do contain undecidable propositions, leading to the Russell’s paradox.

If one builds a sequence for a Turing Machine to describe the phenomena in the universe, the problem of measurement then formally obtains the same representation of a Turing Machine and the Gödel undecidable propositions:

I: logical symbols in term of constants {^~^,∨,∀,0,f,(,),…}.

II: type-1 variables (numbers as quantities, zero included); type-2 variables (classes of quantities); type-3 variables (classes of classes…); type-4(…) variables (classes of…), and so on.

**Definition** **A1.**
*Flg(k) is the set of consequences of k, the smallest set containing all k-formulas, axioms, and is closed with respect to the relationship f of immediate consequence (e.g., in terms of hypersurfaces) (Gödel functions 43,TheoremV) [15].*

**Definition** **A2.**
*a is a sequence of numbers, i.e., a formula.*

**Definition** **A3.**
*ν is the free variable of a.*

**Definition** **A4.**
*Z(n) is the numeral of n with respect to which the proposition is made.*

**Definition** **A5.**
*$Su\;a{(}_{Z(n)}^{\nu })\equiv Sb\left(a_{Z(n)}^{\nu }\right)$ substitute in a of Z(n) to the ν-term of a (Gödel functions 27,30).*

**Definition** **A6.**
*νGena is the generalization of ν with respect to the variable a if the last one is a variable (Gödel function 15).*

**Definition** **A7.**
*Not(x) not-x (Gödel function 13).*

**Definition** **A8.**
*(a) S is ω-non–contradictory (ω-coherent) iff for no property F of natural numbers is it possible to demonstrate if a formula is true or false, i.e., $\left(Ex\right)\;\bar{Fx}$ and all formulas F(i), i=1.*

*(b) k is ω-coherent iff Not(a) such that $\left(n\right)\left[Sb\left(a_{Z(n)}^{\nu }\right)\in Flg\left(k\right)\right]\&\left[Not\left(\nu Gena\right)\right]\in Flg\left(k\right)$ which can be read in these terms: there exists no sequence or sign of class a for which the substitution in a of the numeral of n to the free variable of a can be interpreted as a consequence of the k-propositions and is not a generalization of ν with respect to a.*

**Theorem A1**—following Gödel G-VI [15]

For each class *k* of ω-coherent recursive formulas exist signs of recursive classes *r* such that (νGenr)&(νGenr)~∈Flg(k), i.e., undecidable propositions do exist inside a ω-coherent structure.

**Proposition** **A1.**
*Each formal system S (as Dirac adimensional construction) containing Z with a finite number of axioms and having as inference principles the rule of introduction and that of implication is not complete; there do exist undecidable propositions starting from S axioms, if S is non-contradictory by the following:*

**Proposition** **A2.**
*In S, it is not possible to show that the proposition asserting the non-contradiction of S itself is true or false.*

A formula is demonstrable in a set H when $F_{n}:\sum \left(a_{i}⊃\subset a_{k}\right)$ with 1≤i<k≤n and ∑ be the iterated disjunction ∨ and $a_{j}$ are propositional variables. $F_{n}$ is satisfied by every realization with less than *n* elements, i.e., are satisfied all demonstrable formulas in H. In fact, for each substitution, in at least one of the components for $F_{n},\;a_{i},\;a_{k}$ will be substituted with the same element *e*; and *e*⊃⊂e.∨b for arbitrary *b* gives a privileged value, because *e*⊃⊂e.∨b is H-demonstrable. Taking e.g., the realization $S_{n}$ elements: {1,2,…n}, privileged element 1; a∨b=min(a,b);a∧b=max(a,b);a⊃b=1 if a≥b; and a⊃b=b for a<b;¬a=n for a≠n,¬n=1.

In this case are satisfied by $S_{n}$ all H formulas and $F_{n+1}$ formula with all $F_{j};\;j>n$; but all formulas with smaller j<n values are not satisfied⇒no $F_{n}$ formula is demonstrable in H.

Those theorems also hold for systems with an infinite number of axioms and different inference principles, unless all formulas are sorted and numbered, e.g., by length (etc.) as in a computer database, as well as the classes of numbers associated to axioms. Is also possible to expand the system by introducing some variables for number classes, for classes of number classes, and so on, with their understanding axioms, until reaching transfinite formal systems where theorems hold, and ω-non-contradictoriness in one of those systems is demonstrable inside other bigger systems. Also, non-decidable propositions that demonstrate **p1** become decidable if they are introduced into higher-level structures with their axioms, which are inevitably affected by other undecidable propositions, and so on, as is found in the theory of sets. Building all Gödel construction for the universe, the measure is easily shown to be a *k*-class of ω-coherent propositions in terms of event meta-classes, which contains undecidable propositions inside the structure universe; in fact, all logical structures can be taken back to the above basic formal construction. This is ensured by:

**Theorem** **A1.**

a *(see Gödel [91]) There is no realization with a finite number of elements for which all and only demonstrable formulas in a system of logical propositions H are satisfied, giving privileged values for each substitution.*
b *Between H and the system A of usual propositional calculus, there is an infinite number of systems, i.e., ∃ a decreasing monotonic sequence of a system such that each of them contains H and is contained in A.*

**Lemma** **A1.**
*The measurement process, in the adimensional construction, is a k-class of ω-coherent propositions and contains undecidable propositions.*

**Proof.** *reductio ad absurdum:* Supposing there exists *a* such that $\left(n\right)[Sb\left(a_{Z(n)}^{\nu }\right)\in Flg\left(k\right)]\&$[Not(νGena)]∈Flg(k), it means that substituting for each event *n* its numeral Z(n) to the free variable ν of the sequence of events intended as formulas and measurements, it becomes a direct consequence of those *k* rules (assertions or formulas) without being a generalization of *a* free variables, i.e., without being an extension of the system measure-events. But, during evolution, by state coherence seen above, one must change the structure of the system observer–environment observed [78,79,80], and that written in the symbolic language corresponds to Not(νGena)∉Flg(k)⇒Not(∃)a. □

In fact, a measure gives relationships between other events created by the process itself, i.e., the sequence *a* representing the measurement is a causal sequence of events or coincidence of events that can also belong to the class of “laws”; the evolution of the state universe is a change of the structure universe itself and must be a generalization of the above structure as a self-reproducing fractal one. For this reason, Everett’s Many-Worlds construction is a metastructure for the measurement process and the problem of measurement is translated into the problem of a choice inside a Many-Worlds scenario. The metamathematical structure representing those universes, as happens for every type of formal logic system based on integer numbers, can be translated into a metastructure admitting a splitting that is to be interpreted as true or false. This means that the *M* Rovelli operator [77] is defined and gives a projection of the state “*happened*” or not happened for the universe from all its potential possibilities of existence. Proposition measure and time evolution are decidable only in this metastructure, and the measure “Universe” is seen as a k-proposition justifiable only inside this meta-universe, defined by mutual relationships between subsets of the universe itself. In terms of Gödel numbers, it is represented by the smallest one that defines the next universe chosen between other universe states; in fact, by definition of k-structure, it is the smallest set of information that has all and only that information needed to generate the f− state of the universe that we name “*future*”. All this means that if the structure universe so defined should be logically coherent, it contains some undecidable propositions that can be neither true nor false, suggesting the use of self-bootstrapped models.

It is so possible to build, following those prescriptions, a metastructure in terms of Gödel numerals, and by Church’s lemma, a code for Quantum Turing Machines in the usual Heisenberg representation for quantum computers able to describe the universe inside the Many-Worlds Everett scenario. Time evolution can be set as the problem for self-reproducing a semantically closed cellular automata that evolves, defining space, time, matter and energy as its own aspects, and draws its existence by mutual interaction of its parts, as a self-bootstrapped net does, modifying its own structure during evolution. This implies a self-referential linguistic mechanism, which description is based on symbols related to physical structures or internal states, as a von Neumann automaton [14] does: a self-replicating scheme with memory stored description Φ(A), which can be interpreted by the universal constructor *A* to produce *A* itself; in addition, there is an automaton *B* capable of copying any description Φ included in the self-replication scheme and a third automaton *C* for manipulation of description, ∘. According to Quantum Mechanics prescriptions the initial state *A* must be destroyed in a mixed one with an ancilla *C* to be reproduced in a new state.

The self replicating system is structured into the set of automata (A+B+C) representing the metamathematical description of the universe, and the semantical description Φ(A+B+C)→(A+B+C)∘ is needed to construct the new automaton and describe the new—possible—state of the universe. A system like this, which is able to relate internally stable structures to an interaction with an environmental metastructure, could be seen as a self-reproducing “*organism*” with its semantic closure, and the code mathematically maps “*instructions*” (which are physical actions) into physical actions, to be performed by its composition rules, including emergent physical and linguistic structures.

An alternative description could be given by the self-referential systems that, instead, have different logical constructions that are used for the description of the universe. This was shown with an end-game modeling [64,92,93,94], presenting many properties as a fractal 3- space, derived by universality property; these structures make possible fundamental interaction modeling as a fractal-like structure of emerging spacetime. The description of finite time Quantum Turing Machines corresponds to the use of Heraclitean processes for self-organizing critical systems; start-up axioms are suppressed by requiring that the logic be self-consistently bootstrapped. In this vision, the system moves itself into states characterized by a stochastic process that follow a fractal-like description with no fundamental scales expressed with the language of Wiener processes and shortening all fundamental processes in a very compact way. This is assured by a direct consequence from Gödel conjectures stating that for every formal structure it does exist a metastructure where some undecidable propositions became decidable (logically true or false) and other are described in a more compact way, as the fractal structure assures, employing as main fractal generator the self similarity on the structure given by Heisenberg uncertainty principle. Is also possible to obtain a Gödel code for lie algebras, quantum computer with self referential noise, superspace tools, etc. by Church’s lemma: In some works [95,96] is shown that first order one error quantum computers can give Lie, C*-algebra structures, and a good representation of physical modeling. This corresponds to a finite time computation in a classical Turing Machine given by the finiteness of the quantum of action in Heisemberg uncertainty principle, related to Quantum Turing Machine halting problem [97,98].
